# Sperm parameters and anti-Müllerian hormone remain stable with *Helicobacter pylori* infection: a cross-sectional study

**DOI:** 10.1186/s12894-020-00725-z

**Published:** 2020-11-26

**Authors:** Chun Feng, Ping-Ping Lv, Chang-Chang Huang, Song-Qing Yang, Qiu-Ping Yao, Jin-Ming Shen, Min Jin

**Affiliations:** 1 Department of Reproductive Medicine, The Second Affiliated Hospital of Zhejiang University School of Medicine, 88 Jiefang Road, Hangzhou, 310009 Zhejiang China; 2 The Women’s Hospital of Zhejiang University School of Medicine, Hangzhou, 310006 Zhejiang China; 3 Department of Orthopedics, The First Affiliated Hospital of Zhejiang Chinese Medicine University, 54 Youdian Road, Hangzhou, 310006 Zhejiang China

**Keywords:** *Helicobacter pylori* (HP), Anti-müllerian hormone (AMH), Sperm parameters, Progressive motility

## Abstract

**Background and aims:**

It has been reported that *Helicobacter pylori* (HP) infection was more prevalent in infertile populations. HP infection could lead to decreased sperm parameters, and treating the HP infection could improve the quality of sperm. However, studies investigating the relationship between infertility and HP infection are still limited, and more evidence is required. Therefore, we performed the present study to investigate the impact of HP infection on sperm quality in males and on ovarian reserve in females.

**Methods:**

A total of 16,522 patients who visited the Second Hospital of Zhejiang University from January 2016 to June 2019 due to abdominal discomfort and underwent a ^13/14^C-urea breath HP test were included in this retrospective cross-sectional study. Among them, 565 had performed sperm analysis or ovarian reserve tests in the past three months and were involved for further analyses. Sperm parameters were examined with a computer-assisted sperm analysis system, and serum anti-Müllerian hormone (AMH) and sex hormones were tested with an electrochemiluminescence method.

**Results:**

Among 363 patients who underwent the sperm test, 136 (37.47%) had HP infection. Among 202 patients who underwent the AMH test, 55 (27.23%) had HP infection. There was no difference in sperm concentration and motility between the HP+ and HP− groups (*P* > 0.05). Further subgroup analyses stratified into 5-year age groups confirmed that there was no significant difference in sperm parameters (*P* > 0.05). When pooled with previously published data, no significant difference in sperm concentration or motility was found (*P* > 0.05). Meanwhile, this study found that the serum AMH level was similar between the HP+ and HP− groups (*P* > 0.05). Further subgroup analyses confirmed that there was no significant difference in serum AMH level (*P* > 0.05).

**Conclusions:**

There were no differences in sperm parameters and AMH levels based on history of HP infection among Chinese patients.

## Background

Currently, it is widely accepted that *Helicobacter pylori* (HP) may be related to a series of extragastric diseases, including cardiovascular, neurologic, respiratory, hematologic, metabolic, dermatologic, obstetric, autoimmune, and kidney diseases [1, 2]. Among them, the impact of HP on fertility has attracted much attention. As early as 20 years ago, a study in Italy suggested that the prevalence of HP infection was significantly higher in an infertile population than in controls, and antibodies against HP could be found in follicular fluids, semen, and vaginal secretions [3]. Ten years ago, a study in Japan found that the seropositive rate of HP in an infertile population with unknown etiology was higher than that in a population with known infertility factors, indicating that HP infection could be the cause of infertility [4]. In a cytotoxin-associated gene A (CagA)-positive population, the incidence of early pregnancy loss (EPL) after assisted reproductive technology increased significantly [5]. Recently, studies about infertility have focused on the impact of HP infection on sperm quality.

The first study from Italy reported a lower sperm quality in HP-infected patients with idiopathic infertility than in HP-uninfected patients. In CagA-positive patients, both sperm motility and fertility index are reduced [6, 7]. It has been suggested that anti-CagA antibodies might block spermatozoa acrosomes and disturb fertilization [8]. Further study found that compared with HP− patients, HP+ patients showed reduced sperm concentration, motility, and fertility index [9]. All the above studies indicate that HP infection may be a deteriorating factor for sperm quality, which deserves further investigation and treatment. However, most studies are from Italy, and additional data from different ethnicities may provide more robust evidence.

In females, there is a possible association between HP infection and polycystic ovarian syndrome (PCOS). A study from Turkey reported that the proportion of HP seropositivity was almost doubled in the PCOS population [10]. It is speculated that HP infection may lead to the release of certain substances or stimulate the immune response of the host, leading to the occurrence of PCOS. PCOS is manifested by increased ovarian reserve, while decreased ovarian reserve is an even worse problem that is difficult to treat. Since anti-Müllerian hormone (AMH) is an excellent indicator of ovarian reserve, we plan to investigate the association between HP infection and AMH.

Overall, there appears to be an association between HP infection and infertility, but available support is not sufficient and thus requires further validation. The purpose of the present study is as follows: (1) to investigate the correlation between HP infection and sperm quality in males and (2) to explore the association between HP infection and ovarian reserve in females.

## Methods

### Population of study

**Fig. 1:**
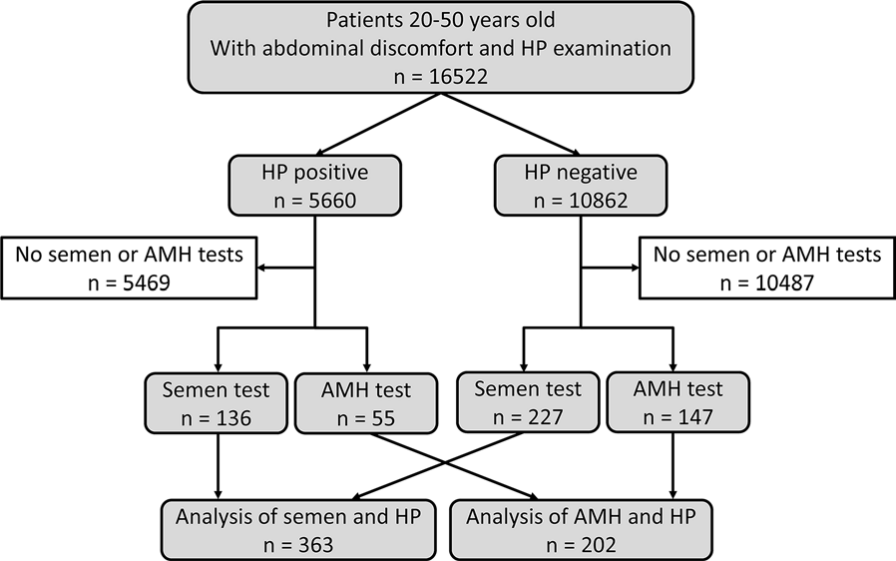
Flow chart of the present study. HP: *Helicobacter pylori*; AMH: anti-Müllerian hormone

From January 2016 to June 2019, patients aged 20–50 years who came to the Second Hospital of Zhejiang University School of Medicine due to abdominal discomfort and underwent HP testing were included in this study. Among them, 565 had plans for pregnancy and had performed sperm analysis or ovarian reserve tests in the past three months, who were involved for further analyses (Fig. 1).

### Detection of HP infection

The ^13^C-urea breath test (UBT) or ^14^C-UBT was used to examine HP infection. Two breath samples were collected before and after ingestion of a ^13^C-urea (Richen–Force, Beijing, China) or ^14^C-urea (Xinke, Shanghai, China) reagent dissolved in water. For the ^13^C-urea breath test, a change over baseline value greater than 4.0 delta over baseline (DOB) was taken as a positive result ( HP+). For ^14^C-UBT, a result greater than 100 DPM was taken as a positive result ( HP+).

### Detection of sperm parameters

Sperm samples were collected with sterile containers by masturbation after 2–7 days of sexual abstinence. After liquefaction at 37 °C for 30 min, routine parameters including sperm concentration and motility were examined with a computer-assisted sperm analysis (CASA) system (WLJY-9000, Beijing, China) according to World Health Organization guidelines [11]. Sperm morphology was assessed by the Papanicolaou staining modified for spermatozoa according to World Health Organization guidelines [11].

### Detection of serum AMH and sex hormones

Serum AMH was tested with the electrochemiluminescence method by an Elecsys ® AMH from Roche Diagnostics on a Roche Cobas e602 analyzer. The total imprecision for the assays was 1.2% at a level of 1.19 ng/mL with a measuring range of 0.01–23 ng/mL. Serum sex hormone levels were detected with the electrochemiluminescence method by kits from Siemens Healthcare Diagnostics Inc.. The total imprecision for the assays was 3.0% at a level of 10,585 pmol/L for estradiol (E_2_), 12.6% at a level of 0.37 nmol/L for testosterone (T), 2.7% at a level of 4.2 IU/L for luteinizing hormone (LH), 3.9% at a level of 6.9 IU/L for follicle-stimulating hormone (FSH), 4.8% at a level of 69.9 mIU/L for prolactin (PRL), and 12.7% at a level of 3.8 nmol/L for progesterone (P). The measuring ranges were 43.6–11,010 pmol/L for E_2_, 0.24–52.05 nmol/L for T, 0.07–200 IU/L for LH, 0.3–200 IU/L for FSH, 6.4–4240 mIU/L for PRL, and 0.67–190.8 nmol/L for P.

### Search strategy and data extraction

To search for studies investigating the correlation between HP and sperm parameters, two reviewers independently searched the studies published in English via three databases, including PubMed, Embase, and Cochrane CENTRAL, until June 30, 2019. Articles were identified through computerized searches using the keywords as follows: ("semen analysis" OR "sperm count" OR "sperm motility") AND ("*Helicobacter pylori*" OR "Campylobacter pylori"). Meanwhile, we hand-searched the references listed in the achieved papers to obtain additional studies.

Two reviewers extracted the common characteristics and outcome parameters of the searched manuscripts independently. The common characteristics included the name of the first author, publication year, country, and number of patients. The clinical outcomes included sperm concentration and progressive motility percentage (PR).

### Statistical analysis

Analyses were performed by using the SPSS 19.0 statistics package (SPSS, Chicago, IL, USA). Continuous variables are expressed as the mean values ± standard deviation (SD). Student’s t test was used for comparisons between two groups. Pearson correlation analysis was performed to analyze the relationship between two variables. A *P* value of < 0.05 was considered statistically significant.

Data from our hospital and previously published results were pooled and calculated together by Review Manager Software (RevMan Version 5.3). When the mean and SD were not provided in the published article, we used formulas to estimate them [12–14]. The results were presented as the mean difference (MD) and 95% confidence interval (CI), and statistical significance was calculated by the Z test. If there was no serious heterogeneity (*P* value ≥ 0.1 by the Q test), a fixed-effects model (FEM) was applied for calculation, and if there was serious heterogeneity, a random-effects model (REM) was applied [15].

## Results

### Baseline characteristics of the involved population.

As shown in Fig. 1, a total of 16,522 patients who underwent the HP test were included in this study. Among these patients, 34.26% (5660) were HP positive. Among the patients with HP infection, 136 underwent the sperm test, and 55 underwent the AMH test. Among the patients without HP infection, 227 underwent the sperm test, and 147 underwent the AMH test. Finally, 363 were involved in the analysis between sperm and HP and 202 between AMH and HP.

As shown in Table 1, the baseline characteristics were similar in both the sperm and AMH analyses. In the analysis of sperm and HP, there was no significant difference in age, weight, height, or body mass index (BMI) between the HP+ and HP− groups (*P* > 0.05). Similarly, in the analysis of AMH and HP, no significant difference was found in age, weight, height, or BMI between the HP+ and HP− groups (*P* > 0.05).

**Table 1:** Characteristics of the present study

Characteristics	Sperm and HP			AMH and HP		
	HP+	HP−	*P*	HP+	HP−	*P*
	n = 136	n = 227		n = 55	n = 147	
Age (y)	31.08 ± 4.28	31.19 ± 4.52	0.822	32.89 ± 6.82	34.27 ± 6.83	0.202
Weight (kg)	69.72 ± 10.05	69.58 ± 9.82	0.898	52.91 ± 8.57	52.85 ± 7.01	0.961
Height (m)	1.74 ± 0.06	1.74 ± 0.06	0.838	1.61 ± 0.05	1.61 ± 0.04	0.546
BMI (kg/m^2^)	22.98 ± 2.78	22.97 ± 2.78	0.967	20.26 ± 2.72	20.39 ± 2.50	0.757
Conc. (Sp/ml × 10^6^)	53.00 ± 42.36	53.90 ± 46.95	0.855	NA	NA	NA
PR (%)	39.39 ± 18.61	39.92 ± 18.81	0.793	NA	NA	NA
Normal (%)	6.73 ± 3.97	6.63 ± 4.43	0.865	NA	NA	NA
Head (%)	86.61 ± 8.71	84.77 ± 13.44	0.248	NA	NA	NA
AMH (ng/ml)	NA	NA	NA	3.49 ± 3.03	3.25 ± 2.82	0.605
E2 (pmol/L)	NA	NA	NA	194.46 ± 78.53	192.56 ± 68.24	0.906
T (nmol/L)	NA	NA	NA	0.94 ± 0.71	1.12 ± 0.78	0.118
LH (IU/L)	NA	NA	NA	6.31 ± 4.32	4.98 ± 3.32	0.106
FSH (IU/L)	NA	NA	NA	8.36 ± 3.84	8.38 ± 3.43	0.978
PRL (mIU/L)	NA	NA	NA	256.19 ± 154.03	234.81 ± 129.36	0.489
P (nmol/L)	NA	NA	NA	1.99 ± 1.03	1.65 ± 0.97	0.128

BMI: body mass index; AMH: anti-Müllerian hormone; NA: not available; Conc.: concentration; PR: progressive motility; Normal: normal sperm morphology percentage; Head: sperm head defects; DFI: sperm DNA fragmentation index; E_2_: estradiol; T: testosterone; LH: luteinizing hormone; FSH: follicle-stimulating hormone; PRL: prolactin; P: progesterone

### Comparison of sperm parameters between groups with or without HP infection

As shown in Table 1, the mean sperm concentration was 53.00 × 10^6^ Sp/mL and 53.90 × 10^6^ Sp/mL in the HP+ and HP− groups, respectively, with no significant difference (*P* > 0.05). Sperm PR was also similar between the HP+ and HP− groups (39.39% vs. 39.92%), with no significant difference (*P* > 0.05). There was no difference in either normal sperm morphology percentage or sperm head defects (*P* > 0.05).

**Fig. 2:**
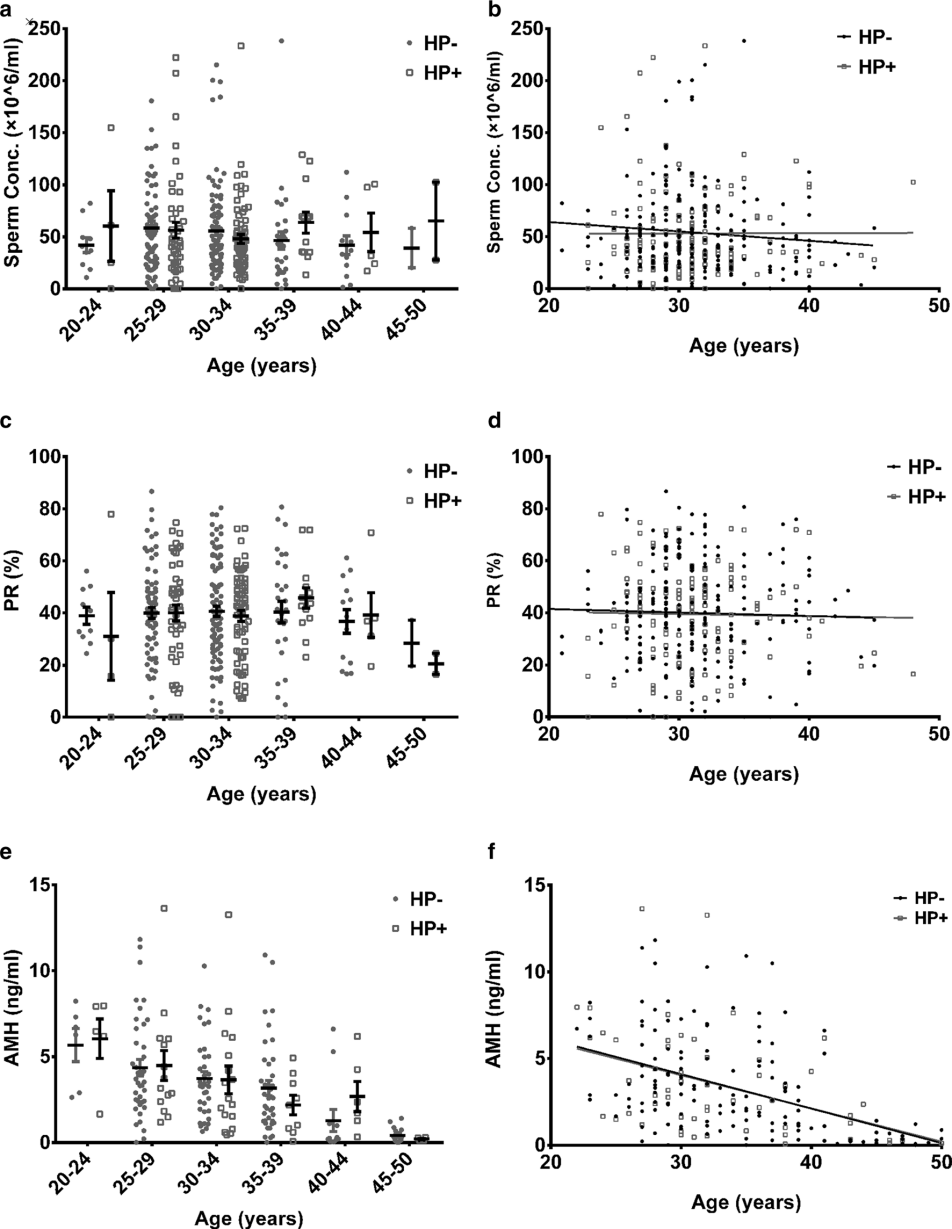
Correlation between sperm parameters and anti-Müllerian hormone (AMH) and *Helicobacter pylori* (HP) infection. **a**, **b** Correlation between AMH and HP. **c**, **d** Correlation between sperm concentration (Conc.) and HP. **e**, **f** Correlation between progressive motility percentage (PR) and HP

To further exclude the impact of age, we divided the population into subgroups of 20–24, 25–29, 30–34, 35–39, 40–44, and 45–50 years of age. As shown in Fig. 2a, c, there was no significant difference in sperm concentration or PR between the HP+ and HP− groups for any age group (*P* > 0.05). As shown in Fig. 2b, d, in both the HP+ and HP− groups, there was no significant correlation between sperm concentration and age or between PR and age (*P* > 0.05).

### Comparison of AMH and sex hormones between groups with or without HP infection

As shown in Table 1, the mean serum AMH level was 3.49 in the HP+ group and 3.25 in the HP− group, with no significant difference (*P* > 0.05). No difference was found between HP+ and HP− groups in serum E_2_, T, LH, FSH, PRL, or P levels (*P* > 0.05).

As shown in Fig. 2e, there was no significant difference in AMH level between the HP+ and HP− groups in every age span (*P* > 0.05). Meanwhile, AMH correlated significantly negatively with age (Fig. 2f, for HP−, Pearson correlation coefficient = − 0.482, *P* = 0.000; for HP+, Pearson correlation coefficient = − 0.431, *P* = 0.001).

### Pooled analysis of the association between sperm parameters and HP infection

Since 2010, six studies investigated the correlation between HP infection and sperm parameters, as listed in Table 2. Most studies found that sperm motility was reduced significantly in CagA+ patients [6, 7, 16, 17]. The latest study found that sperm concentration and PR were reduced in the HP+ population, and in the CagA+ population PR was reduced further than in the CagA− population [9].

**Table 2:** Previous studies investigating the impact of HP infection on sperm parameters

Study	Public year	Country	HP (+ vs. −)			CagA (+ vs. −)			Results
			Population	Conc	PR (%)	Population	Conc	PR (%)	
Moretti [9]	2017	Italy	32 vs. 41	38.0 vs. 55.0*	17 .0 vs. 34.0***	20 vs. 12	33.4 vs. 42.5	10.5 vs. 22.5***	HP+ reduced conc. and PR
Moretti [17]	2015	Italy	28 vs.81	61 vs. 72	32 vs. 32	12 vs. 16	61 vs. 61.5	24 vs. 36.5*	CagA+ reduce PR
El-Garem [18]	2014	Egypt	NA	NA	NA	22 vs. 201	NA	NA	HP treatment improved PR
Moretti [16]	2013	Italy	NA	NA	NA	37 vs. 50	58 vs. 63	18 vs. 32**	CagA+ reduced PR
Moretti [7]	2012	Italy	27 vs. 51	94 vs. 72	32 vs. 30	11 vs. 16	65 vs. 94	24 vs. 38**	CagA+ reduced PR
Collodel [6]	2010	Italy	36 vs.44	24.5 vs. 23.5	22 vs. 28.5	17 vs. 19	25.5 vs. 23	18 vs. 29*	CagA+ reduced PR

NA: not available; Conc.: sperm concentration; PR: progressive motility; CagA: cytotoxin-associated gene A

^*^P < 0.05; **P < 0.01; ***P < 0.001

**Fig. 3:**
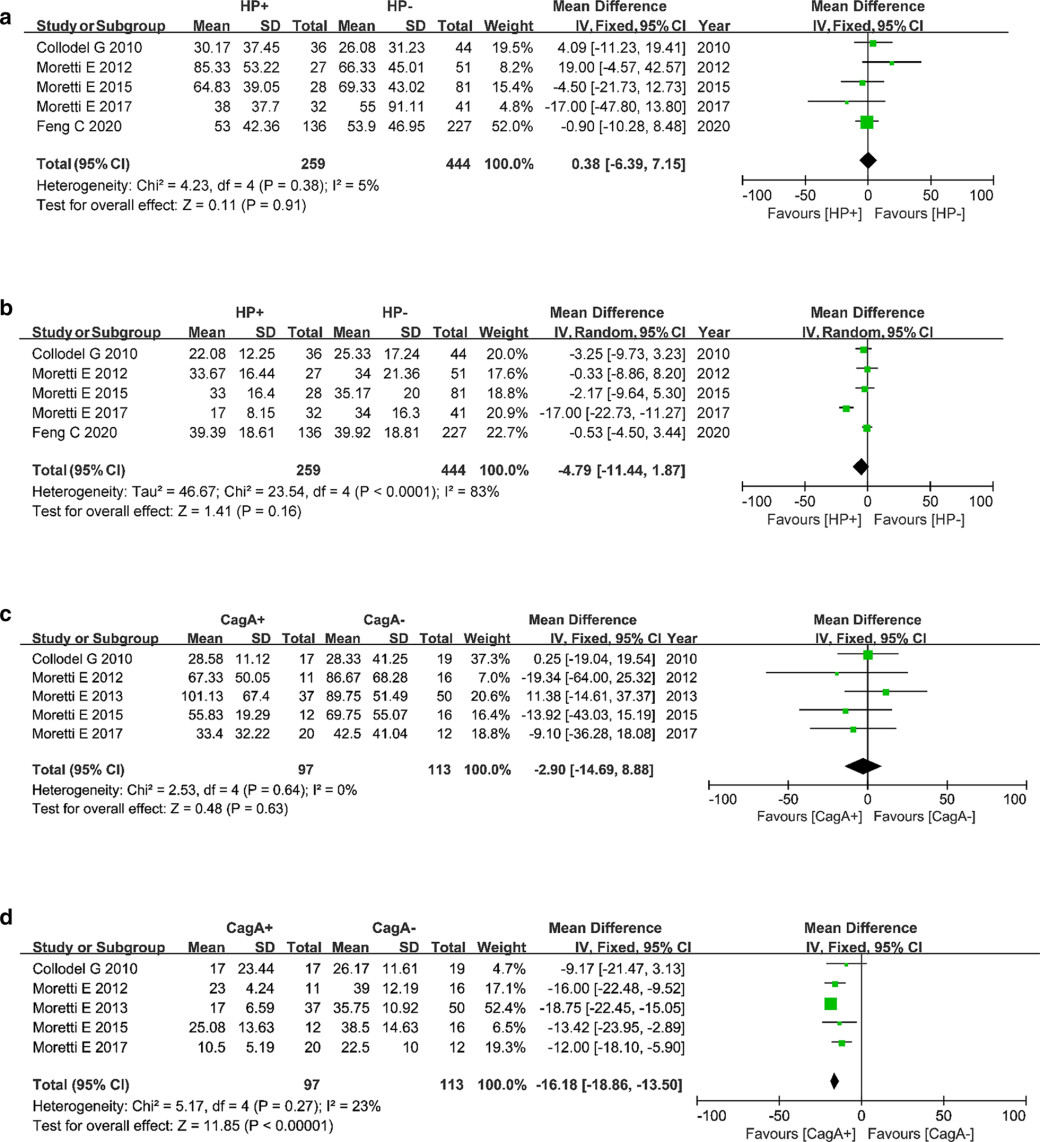
Forest plot of the association between *Helicobacter pylori* (HP) infection and sperm parameters. **a** Sperm concentration and HP infection. **b** Progressive motility percentage (PR) and HP infection. **c** Sperm concentration and cytotoxin-associated gene A (CagA) strains. **d** PR and CagA strains

Five studies including 703 participants were pooled to compare HP+ and HP− groups, and five studies including 210 participants were pooled to compare CagA+ and CagA− groups. As shown in Fig. 3a, c, FEM analysis showed that there was no significant difference in sperm concentration between the HP+ and HP− groups or between the CagA+ and CagA− groups (*P* > 0.05 for both). In the sperm motility analysis between HP+ and HP−, since serious heterogeneity (*P* < 0.01) was found, a REM was applied and suggested no significant difference in PR (Fig. 3b, 95% CI − 11.44 to 1.87, *P* = 0.16). FEM analysis was applied to compare sperm PR between CagA+ and CagA− groups, which suggested that PR was 16.18% lower in the CagA+ group than in the CagA− group (Fig. 3d, 95% CI − 18.86 to − 13.50, *P* < 0.01).

## Discussion

In the present study, no difference was found in sperm concentration or sperm motility between HP+ and HP− groups. Further subgroup analyses confirmed that there was no significant difference in sperm parameters between HP+ and HP− groups. Furthermore, we pooled our data and those of previous studies and found no significant difference in sperm concentration or motility, indicating that in the Chinese population, HP infection does not disturb spermatogenesis.

The results of previous studies were not consistent. Some suggested that sperm concentration and motility were reduced in HP+ patients [9] and that treating HP could improve the quality of sperm [18], while some suggested no significant difference in sperm parameters between HP+ and HP− groups [6, 7, 16, 17]. The inconsistent results may be due to different test methods and ethnicities investigated.

This is the first study that used UBT to detect HP infection and to investigate its relationship with sperm quality. In previous studies, HP infection was detected with a serology test by enzyme-linked immunosorbent assay (ELISA) and confirmed with western blotting (WB) [6, 7, 9, 16–18], whereas in the present study, ^13^C- and ^14^C-UBT were used to detect HP infection. UBT is the best noninvasive method for patients without gastric resection or proton pump inhibitor (PPI) treatment, with both high positive predictive value and negative predictive value [19–21]. A meta-analysis suggested that UBT had high diagnostic accuracy for detecting HP infection in patients with dyspepsia, with a pooled sensitivity of UBT in adult patients of 96% and a pooled specificity of 93% [22, 23]. Another meta-analysis involving 34 studies with serology evaluation and 57 studies with UBT detection reported that the sensitivity of HP diagnosis was 0.94 for ^13^C-UBT, 0.92 for ^14^C-UBT, and 0.84 for serology tests. UBT showed a higher diagnostic accuracy than the serology test for HP infection diagnosis [24, 25]. Therefore, in this study, UBT was used, as it provides a more accurate HP diagnosis than serology tests.

Moreover, serology tests cannot distinguish between active and inactive infections [26]. In a letter from Caviglia et al., the authors emphasized that the presence of serological HP antibodies could only indicate previous exposure, not necessarily a current infection, and based on this, they recommended UBT as a direct diagnostic test [27]. Similarly, in the present study, UBT examination represented the status of current HP infection better than serology tests.

CagA is the major virulence factor in HP, encoding the CagA protein in the cag pathogenicity island [28]. HP infection can be divided into two isolates: CagA-producing strains (CagA+) and CagA-nonproducing strains (CagA−). Our meta-analysis of sperm motility and CagA-producing/nonproducing strain infection suggested that PR was 16.18% lower in the CagA+ group than in the CagA− group. The underlying mechanism may be that CagA+ HP infection induces overexpression of miR-543 and downregulation of the p14ARF tumor suppressor to inhibit autophagy and increase cytokine production, which induces inflammatory responses of HP accordingly [29–31]. Anti-CagA antibodies may block spermatozoa acrosomes and disturb fertilization [8].

The prevalence of the CagA genotype in HP infection varies significantly among different regions. In Western countries, CagA+ strains comprise 50–60% of the HP+ population, and in the Chinese population, CagA+ strains occupy nearly 100% of the HP+ population [32, 33]. Studies investigating the CagA status of Chinese HP strains with polymerase chain reaction (PCR) detected CagA genotypes in nearly all strains [34, 35]. Considering the high CagA positivity in the Chinese HP+ population, the sperm concentration and motility should be weakened in HP+ patients, but the present study showed stable parameters. Further study investigating CagA antibody status should be performed to clarify the role of CagA in sperm quality.

The variability of sperm parameters after HP treatment is an interesting question. It was reported that after the treatment of HP, seminal HP IgA level decreased significantly, and meanwhile progressive sperm motility, nonprogressive sperm motility, and sperm normal forms increased significantly (*P* = 0.001) [18]. In the present study, sperm analyses were performed before HP test, and most patients with HP+ suspended their plans of pregnancy after HP treatment. Therefore, we did not follow the sperm parameters.

In the present study, there was no difference in serum AMH level between HP+ and HP− groups, which was confirmed with further age-divided subgroup analyses. Published results of the relationship between PCOS and HP infection are inconsistent. Yavasoglu et al. found that HP antibody positivity was significantly more common in the PCOS group than in the age-matched control group [10]. The possible explanation may be that the antigenic mimicry to HP antigens leads to an immune cross-reaction between HP antigens and the ovaries, inducing the onset of PCOS [36]. Nevertheless, Tokmak et al. found no significant difference in HP IgG positivity between PCOS and non-PCOS groups [37]. AMH is a potential future substitute for detecting polycystic ovarian morphology (PCOM) and a useful biomarker for predicting the risk of PCOS [38–40]. Our data indicated no correlation between PCOS and HP infection. Meanwhile, AMH is considered the best serum biomarker of ovarian reserve, reflecting the number of primordial follicles and its response to exogenous gonadotropins [41]. The present study indicates that ovarian reserve is stable with HP infection.

## Conclusion

This is the first observation investigating the impact of HP infection on ovarian reserve, which found that HP infection was not related to the serum ovarian reserve biomarker AMH. In general, HP infection is not a crucial factor affecting sperm parameters or ovarian reserve.

## Data Availability

The data analyzed in this study are available from the corresponding author upon request.

## Abbreviations

DOB: Delta over baseline
E_2_: Estradiol
EPL: Early pregnancy loss
FEM: Fixed-effects model
FSH: Follicle-stimulating hormone
HP: *Helicobacter pylori*
LH: Luteinizing hormone
MD: Mean difference
P: Progesterone
PCOM: Polycystic ovarian morphology
PCOS: Polycystic ovarian syndrome
PCR: Polymerase chain reaction
PPI: Proton pump inhibitor
PR: Progressive motility percentage
PRL: Prolactin
REM: Random-effects model
SD: Standard deviation
T: Testosterone
UBT: Urea breath test
WB: Western blotting
