# Correction to: Sperm parameters and anti-Müllerian hormone remain stable with Helicobacter pylori infection: a cross-sectional study

**DOI:** 10.1186/s12894-021-00783-x

**Published:** 2021-02-05

**Authors:** Chun Feng, Ping-Ping Lv, Chang-Chang Huang, Song-Qing Yang, Qiu-Ping Yao, Jin-Ming Shen, Min Jin

**Affiliations:** 1 Department of Reproductive Medicine, The Second Afliated Hospital of Zhejiang University School of Medicine, 88 Jiefang Road, Zhejiang, 310009 China; 2 The Women’s Hospital of Zhejiang University School of Medicine, Hangzhou, 310006 Zhejiang China; 3 Department of Orthopedics, The First Affliated Hospital of Zhejiang Chinese Medicine University, 54 Youdian Road, Hangzhou, 310006 Zhejiang China

## Correction to: BMC Urol (2020) 20:188 https://doi.org/10.1186/s12894-020-00725-z

It was highlighted that the original publication erroneously contained the galley proof instead of the final version of the article. The Publisher would like to apologize to the authors and readers for the inconvenience.
